# Methylation entropy as a novel dimension in liquid biopsy: enhanced multimodal framework for cancer detection and tissue-of-origin classification

**DOI:** 10.1186/s12967-026-08028-x

**Published:** 2026-03-19

**Authors:** Xiaoqiong Jia, Yongjun Li, Baochen Du, Jie Zhao, Xiuxiu Wang, Hong Yang, Qiang Zhao, Qin Si, Yuanyuan Guo, Bateer Han, Zhenghang Wang, Junqing Liang, Yongfei Peng, Shuanping Liu, Guangpeng Zhou, Zhongwu Li, Ziyu Li, Xiangdong Fang, Xiaoliang Han

**Affiliations:** 1 Peking University Cancer Hospital (Inner Mongolia Campus)/Affiliated Cancer Hospital of Inner Mongolia Medical University, Hohhot, China; 2 BioChain (Beijing) Science & Technology, Inc., Beijing, China; 3 China National Center for Bioinformation, Beijing Institute of Genomics, Chinese Academy of Sciences & University of Chinese Academy of Sciences, Beijing, China; 4 Peking University Cancer Hospital & Institute, Beijing, China

**Keywords:** Methylation entropy, Multimodal model, Multi-cancer detection, cfDNA, Non-invasive liquid biopsy, Early-cancer detection

## Abstract

**Background:**

Circulating cell-free DNA (cfDNA) is a promising biomarker for non-invasive early cancer detection. We integrated methylation entropy, a measure of DNA methylation heterogeneity, with other cfDNA features into a multimodal model to improve multi-cancer detection.

**Methods:**

A customized panel named HYGEIA, containing abundant informative methylation CpG sites, was first developed. The panel’s performance was validated, and a method for calculating fragment-level methylation entropy was established. Subsequently, this panel was utilized to perform deep panel-targeted bisulfite sequencing on 521 plasma samples from 288 cancer patients and 233 non-cancer individuals. Participants were randomly allocated into training (197 cancer, 157 non-cancer) and testing (91 cancer, 76 non-cancer) sets for constructing the Enhanced Multi-Cancer Early Detection (EMCED) model. Upon validation of the EMCED model, a Tissue-of-Origin (TOO) model was further developed and evaluated across seven cancer types.

**Results:**

A classifier based solely on methylation entropy achieved an AUC of 0.938 (sensitivity 78.0%, specificity 94.7%) in the testing set for multi-cancer detection. Building on this, two additional characteristics, DNA methylation levels and fragmentation features, were integrated into the model, resulting in the EMCED model, which achieved an AUC of 0.979 (sensitivity 93.4%, specificity 93.4%) in the testing set. Analysis of the EMCED model’s sensitivity by cancer type showed detection rates of 95.5% for colorectal cancer, 98.0% for lung cancer, 93.5% for gastric cancer, 83.8% for ovarian cancer, and 100% for liver, esophageal, and thyroid cancers. The TOO model, which incorporated all three feature types, methylation entropy, methylation levels, and fragmentation features, achieved an accuracy of 92.3% for classifying the tissue of origin among cancer cases in the testing set.

**Conclusions:**

Methylation entropy is a robust cfDNA biomarker for distinguishing cancer from non-cancer. The multimodal model enables enhanced multi-cancer detection and accurate classification, supporting the integration of methylation entropy for improved non-invasive early cancer detection.

**Supplementary Information:**

The online version contains supplementary material available at 10.1186/s12967-026-08028-x.

## Background

As a major global disease, cancer has been one of the leading causes of death worldwide, as well as a public health issue that poses a serious threat to human health [1]. Previous study has indicated that early detection and timely diagnosis can improve the survival rates of cancer patients [2]. Moreover, compared to single-cancer screening, which often suffers from low compliance due to the time and cost associated with repeated screening for cancer types, multi-cancer screening has been demonstrated to effectively improve participation rates and early detection efficiency by enabling the screening of multiple cancers in a single test [3]. Multi-cancer early detection (MCED) allows the detection of signals from multiple cancers through a single test and has the potential to identify cancers for which no current screening or early detection strategies exist [4]. Given the complexity of the mechanisms underlying different cancers, relying solely on a single type of biomarker or a simple model is insufficient to comprehensively and accurately detect cancer signals and predict cancer types. Therefore, the combination of multiple biomarkers and the integration of various predictive models hold promise as effective approaches for advancing multi-cancer early screening and detection.

In recent years, circulating cell-free DNA (cfDNA) has attracted attention for its applications in non-invasive detection, with cfDNA methylation profiling emerging as a critical biomarker for cancer screening. Researchers have made substantial efforts in technological innovation and model development to improve the performance of cfDNA methylation in cancer detection. Leveraging the high-throughput capabilities of next-generation sequencing (NGS), several multi-cancer screening studies, such as Galleri, CancerSEEK, ELSA-seq, THEMIS, and Panseer, have achieved remarkable results, most of which rely on the analysis of DNA methylation features [5–9]. Additionally, many preclinical studies utilized public databases, such as TCGA 450 K and GEO, to identify differentially methylated sites, which were then combined with in-house data to construct panels for clinical validation [10, 11]. Although the use of methylation panels for cancer detection has made notable progress, the accuracy of multi-cancer detection in these studies still requires further improvement. Considering the shared methylation characteristics of nearly all cancer types, hypomethylation in low-density CpG regions and hypermethylation in CpG islands, researchers believe that deep sequencing of these regions can help identify effective diagnostic biomarkers [12], thereby enabling the development of more accurate identification models for multiple cancer types.

Recent epigenetic research into DNA methylation has introduced the concept of methylation entropy. In the field of thermodynamics, entropy is a quantification concept describing the level of disorder in a system. In the context of disease inspection, particularly in cancer, variability in methylation states among cells, DNA methylation heterogeneity, has been recognized as an important indicator of disease progression [13]. In 2011, Xie, et al. were the first to apply Shannon Entropy as a measure of DNA methylation heterogeneity across genomic sites, revealing that tumor tissues exhibit higher methylation entropy compared to normal tissues [14]. Similarly, Ramakrishnan et al. employ entropy to analyze DNA methylation features of genes associated with Kidney Renal Clear Cell Carcinoma (KIRC), discovering this calculating approach could enhance our understanding of cancer-specific methylation patterns [15]. Moreover, several studies have utilized methylation entropy to investigate the correlation between individual methylation characters and aging, demonstrating that methylation entropy increases with age and linking aging to a more chaotic methylation landscape [16–18]. These findings collectively underscore the potential utility of methylation entropy as a diagnostic, screening, or health-management tool in the contexts of disease and aging.

In this study, we introduced the concept of methylation entropy to explore differences in methylation patterns between cancer patients and non-cancer individuals. Using panel-targeted sequencing data of cfDNA, we calculated methylation entropy values and developed a model for multi-cancer detection. The detection performance of the methylation entropy model was evaluated and demonstrated outstanding effects in distinguishing cancer patients from non-cancer individuals. Additionally, we optimized the model by integrating methylation levels and fragmentation features to create a multimodal model, which further improved the sensitivity and specificity of multi-cancer detection. Moreover, a Tissue-of-Origin model was developed to classify the cancer type in patients, covering seven cancers: lung, colorectal, gastric, liver, esophageal, thyroid, and ovarian cancers.

## Participants and Methods

### Study design

This study is a single-center, case-control study designed to develop early detection methods for seven cancer types, colorectal, lung, gastric, ovarian, liver, esophageal, and thyroid, using cfDNA. The study was divided into three steps. In the first step of the study, we introduced a customized panel, the HYGEIA panel, to perform targeted bisulfite sequencing on clinical samples, validated the panel, and calculated fragment-level methylation entropy. In the second step, we constructed the binary classification model utilizing cfDNA methylation data to distinguish cancer patients from non-cancer individuals and performed model optimization and evaluation. In the third step, the Tissue-of-Origin (TOO) model was constructed and its accuracy for the top 1-predicted (TOP1) origin and top 2-predicted (TOP2) origins was evaluated. The primary outcomes were sensitivity, specificity of the binary classification model, and TOP1 accuracy and TOP2 accuracy of TOO model.

### Clinical participants

All clinical samples used in this study were derived from Affiliated Cancer Hospital of Inner Mongolia Medical University. This study was approved by The Ethics Committee of Peking University Cancer Hospital (Inner Mongolia Campus)/Affiliated Cancer Hospital of Inner Mongolia Medical University. The ethical approval numbers are as follows:

*The Ethics Committee of Peking University Cancer Hospital (Inner Mongolia Campus)/Affiliated Cancer Hospital of Inner Mongolia Medical University KY202401*.

All procedures were in accordance with the ethical standards of the responsible ethics committee and with the Helsinki Declaration of 1964 and later versions. Informed consent was obtained from all patients before sample collection.

In the first step of this study, a total of 74 tissue samples were collected, including tumor tissues and adjacent non-tumor tissues from patients with seven distinct cancer types. Each cancer tissue sample and its corresponding non-cancer (adjacent normal) tissue sample were obtained from the same individual. In the second and third steps, which involved the development of the binary classification model and the tissue-of-origin classification model, a case-control cohort was employed, comprising 521 participants in total. Blood samples were collected from the 521 participants. Each blood sample in this case-control cohort was from a different participant. This cohort included 288 cancer patients (covering seven cancer types: colorectal, esophagus, liver, gastric, lung, ovarian, thyroid) and 233 non-cancer controls.

#### Inclusion and exclusion criteria

1. Participants with baseline clinical characteristics and successful collection and extraction of plasma cell-free DNA (cfDNA) were enrolled.
2. Blood samples for cfDNA extraction were prospectively collected from patients prior to surgical intervention and before a definitive cancer diagnosis. Subjects with a high suspicion of cancer based on clinical and/or imaging assessment, such as CT revealing tumors of undetermined malignancy, provided informed consent and underwent blood collection for cfDNA methylation analysis prior to a planned biopsy or surgical resection.
3. Pathological examination of surgically resected tissue served as the gold standard for definitive diagnosis of cancer. Patients with confirmed malignancy were classified as the cancer group. Each pathological examination result of cancer patients was evaluated and confirmed by a senior pathologist.
4. Cancer patients who had received chemotherapy, radiotherapy, immunotherapy, or any other cancer treatment prior to blood sample collection were excluded.
5. Patients diagnosed with more than one type of primary cancer were excluded. Specifically, beyond confirmation of cancer diagnosis using the gold standard methods, each patient also underwent imaging examinations and routine physical assessments and was evaluated by a senior clinical expert to ensure that, apart from the cancer-affected organ, there were no significant diseases present in other organs or systems.
6. Non-cancer participants were individuals who had abnormal medical findings. After obtaining informed consent, blood samples were collected from these individuals who were later confirmed not to have cancer through imaging or other examinations, as assessed by a senior clinical expert.

### Prospective analysis

The study was conducted in a prospective and blinded manner. Specifically, an independent moderator supervised the entire process to ensure strict separation between laboratory, clinical, and analytical teams. After blood collection, laboratory personnel extracted cfDNA and performed panel-targeted sequencing without access to any clinical information. Meanwhile, clinical experts managed patient diagnosis and follow-up (biopsy and pathological confirmation) without access to sequencing data. The moderator subsequently compiled the clinical information, performed data labeling, and assigned samples to training and testing sets before transferring the datasets to the analytical team. Importantly, the data analysts involved in model development were blinded to the true clinical information throughout the analysis process. The moderator then unblinded the results only after modeling had been completed.

### Panel design

To maximize the capture of cfDNA signals and minimize the risk of missing critical information, we screened DNA methylation data from multiple sources to select regions or loci for analysis. For the initial screening, we primarily utilized in-house WGBS data from both tissue and plasma cfDNA. Differential methylation analysis was performed between carcinoma tissues and adjacent normal tissues, as well as between cfDNA samples from cancer patients and non-cancer individuals. The candidate DNA regions, significantly differentially methylated positions (DMPs), identified from these differential analyses were then further filtered by assessing background methylation levels using WGBS data from white blood cells (WBCs). Regions exhibiting high background methylation in WBCs were excluded from further consideration. In addition, we incorporated differentially methylated positions (DMPs) identified from publicly available datasets, including data from The Cancer Genome Atlas (TCGA) and the Gene Expression Omnibus (GEO). Specifically, we included DMPs derived from the Infinium Human Methylation 450 K Array in TCGA, covering seven cancer types (lung, colorectal, gastric, liver, esophageal, thyroid, and ovarian cancer; with cancer tissue *n* = 2,602, adjacent/normal tissue *n* = 234). Furthermore, we analyzed WGBS data for lung, colorectal, liver, and esophageal cancers obtained from GEO (GSE79799, GSE70090, GSE46644, GSE137879, GSE149608; cancer tissue *n* = 24, adjacent/normal tissue *n* = 29).

Based on the inclusion and exclusion of DMPs identified from the above analyses, we constructed a customized panel, the HYGEIA panel, encompassing 32 Mb, which includes 99% of CpG islands (CGIs; 27,718 CGIs) as well as other relevant genic regions. The HYGEIA panel involves more than 19,000 genes, which include oncogenes, proto-oncogenes, tumor suppressor genes, immune-related genes, methylation-related genes, and metabolism-related genes.

### Blood sample collection and cfDNA preparation

Peripheral blood (8–10 mL per assay) was collected from each participant using specialized blood collection tubes. Plasma cfDNA was extracted using the QIAamp Circulating Nucleic Acid Kit (QIAGEN, Cat. No. 55114). The extracted cfDNA was quantified and then subjected to bisulfite conversion using the EZ DNA Methylation-Gold™ Kit (Zymo Research, Cat. No. D5005, Irvine, CA) according to the manufacturer’s protocol.

### Library construction and panel-targeted sequencing

After bisulfite conversion, cfDNA was used for the preparation of dual-indexed sequencing libraries. Library construction was performed using the HiLCS (High-Depth Library Construction System) method based on Splinted Adaptor Tagging (SPLAT), enabling highly efficient library generation from single-stranded bisulfite-converted DNA [19]. Target enrichment was achieved through liquid-phase hybridization capture using a custom-designed probe panel (HYGEIA) that targets CpG sites informative for multi-cancer detection [20]. Biotinylated DNA probes complementary to the target regions were hybridized to the prepared libraries, and the hybridized DNA–probe complexes were captured using streptavidin-coated magnetic beads. After stringent washing to remove non-specific fragments, the enriched target DNA was PCR-amplified and subjected to high-depth sequencing on an Illumina platform.

### Bioinformatics algorithms

#### MethBin segmentation

DNA segments were defined using MethBin. MethBin merges adjacent regions in thehuman genome that are less than 400 bp apart and contain at least three CpG sites to generate target segments for downstream analysis. These segmented regions were subsequently used for the calculation of fragment-level methylation entropy and methylation level.

#### Methylation entropy calculation

The cfDNA or tissue DNA sequences captured using the HYGEIA panel were mapped to regions partitioned by MethBin, which may consist of multiple discontinuous captured segments. Each of these segments is referred to as an Inserted Fragment. For each Inserted Fragment, we applied the following filtering criteria: (1) fragments with fewer than three or more than 32 CpG sites were excluded; (2) fragments with missing CpG sites in the middle of the sequence were also excluded. After filtering, methylation entropy for each qualified Inserted Fragment was calculated using the BiEntropy algorithm [21] according to the following formula:

$$\begin{aligned}BiEn(s)&=(1/(2^{n-1}-1))\cr&\left(\begin{aligned}&\sum_{k=0}^{n-2}((-p(k)log_2\:p(k)\cr&-(1-p(k))log_2\:(1-p(k))))2^k\end{aligned}\right)\end{aligned}$$

Subsequently, we calculated the fragment-level methylation entropy for a given MethBin region by the formula:

$$\rm Entropy\:value = 1/N * (e1 + e2 +e3 +\:...+eN)$$

where N is the total number of Inserted Fragments within a given MethBin region, and e is the BiEn(s) of each Inserted Fragment calculated using the BiEntropy algorithm. A Python package, BiEntropy (version 1.1.4), was used in this analysis. The code for methylation entropy analysis has been uploaded to GitHub.

#### U reads abundance (URA)

For each target region partitioned by MethBin, only sequencing reads containing at least four CpG sites were retained. The methylation level u for each read was calculated as the proportion of methylated CpG sites among all CpG sites, i.e., $u=\frac{C}{C+T}$, where C is the number of methylated CpG sites and T is the number of unmethylated CpG sites. Reads were classified as U reads (mostly unmethylated) if $u\le\frac{1}{4}$, M reads (mostly methylated) if $u\ge\frac{3}{4}$, and X reads (mixed methylated) if $\frac{1}{4}<u<\frac{3}{4}$. For each target interval, U reads abundance (URA) was calculated as the proportion of U reads among all reads in that interval, i.e., $\frac{U}{U+X+M}$. To ensure robust quantification, a minimum of 50 effective reads per region was required, i.e., $U+X+M\ge50$.

#### Fragmentation

The hg19 assembly was used as the human reference genome. The autosomes of the reference genome were segmented into adjacent, non-overlapping 100 kb bins. Low-mappability bins were filtered according to Cristiano et al. [22], excluding the 10% of bins with the lowest coverage and removing reads mapping to Duke blacklist regions (http://hgdownload.cse.ucsc.edu/goldenpath/hg19/encodeDCC/wgEncodeMapability/*).* To reduce feature dimensionality and noise, neighboring bins were subsequently merged into 1 Mb region. The captured intervals of cfDNA were classified by length as follows: short (100–150 bp), middle (150–260 bp), and long (260–320 bp). The coverage of short, middle, and long intervals was calculated respectively for each 1 Mb region. Then the sum of three kinds of coverage was calculated, denoted as total fragmentation coverage (nfrags). Code section reference: https://github.com/Cancer-Genomics/delfi_scripts.

### Model construction

#### Data preprocessing

For the methylation entropy and methylation models, we excluded regions partitioned by MethBin with a sequencing depth of less than 50 or with missing methylation entropy values (NA) in more than 90% of samples. No depth filtering was applied to the fragmentation model.

#### Training and testing sets

There were 521 participants in total, including 288 cancer patients and 233 non-cancer individuals. Among them, the training set contained 197 cancer patients and 157 non-cancer individuals, and the testing set contained 91 cancer patients and 76 non-cancer individuals. The clinical information and case numbers of the seven cancers in the training and testing sets are presented in Table 1 and Supplementary Table S1. Feature selection was then performed using only the training set. Subsequent model training was also based on the samples in the training set. After the models were trained and fixed, the testing set was used for performance evaluation.

#### Cancer signal detection model

##### Feature selection

Data from all cancer types in the training set were merged as the case group, while non-cancer individuals in the training set constituted the control group. For each MethBin region, sensitivity, methylation difference (delta), and P-value were calculated under the condition of 80% specificity. The selection criteria were as follows: absolute value of delta (|delta|) ≥ 0.02, sensitivity ≥ 0.4 (for the methylation entropy model) or ≥ 0.5 (for the methylation model), and *P* < 0.01. Subsequently, candidate features were further filtered using the random forest algorithm (Python package, sklearn, version 1.2.1) with a feature importance threshold > 0.006 (for the methylation entropy model) or > 0.0055 (for the methylation model). Ultimately, 19 features were retained for methylation entropy model, and 32 features were retained for methylation model.

For the fragmentation model, the features are based on coverage values derived from the fragmentation algorithm described above. Specifically, for each 1 Mb region across the whole genome, four types of coverage are calculated: coverage of short intervals, coverage of middle intervals, coverage of long intervals, and total coverage. These coverages across all regions were used as features for each sample. Principal component analysis (PCA) was then performed on them, and the resulting principal components for each sample were used as the input features for training the fragmentation model.

##### Model training and testing

Binary classification models were constructed using logistic regression (Python package, sklearn, version 1.2.1) in the training set and validated in the testing set. For the fragmentation model, the classifier was built using elastic net regression based on the top 98% principal components derived from the training set. The R package *caret* (version 6.0.93) was employed in this step.

#### Multi-cancer tissue-of-origin model

##### Methylation entropy model

For each of the seven cancer types, binary analyses were performed comparing each single cancer type against all others (at 80% specificity) in order to identify cancer-type-specific markers. The combined markers were further refined using the Boruta algorithm (R package, Boruta, version 8.0.0), resulting in a final set of 107 features. The gender variable was also included as a feature for ovarian cancer. The final classification model was established using logistic regression.

##### Methylation model

Based on URA features, candidate regions were first selected using the Kruskal-Wallis test (with FDR correction, q < 0.05). Pairwise comparisons of candidate regions between cancer types were then performed using the Dunn’s test to identify cancer-type-specific regions. For each cancer type, the top 30 features with the highest contribution as determined by the Boruta algorithm (a total of 195 features) were selected; the gender variable was also included. The final classifier was constructed using elastic net regression.

##### Fragmentation model

The classification model was built using elastic net regression based on the top 98% principal components from the training data, together with the gender variable.

The schematic of the model-construction pipeline is presented in the Supplementary Figure S2.

### Simultaneous analysis based on real-world data

The interception model was employed to evaluate the real-world benefit of AKSO in clinical settings [23]. The core concept of the interception model is that the development and progression of all cancers universally proceed through stages I, II, III, and IV. For the AKSO model, the number of individuals detected at each stage comprises two components: (1) newly detected individuals at the current stage, and (2) individuals who progressed from the previous stage without being detected (i.e., individuals who “slipped” from the earlier stage). The formula is as follows:

Detectable at stage = (original individuals) * (marginal sensitivity) + (individuals slipped from earlier stage).

Marginal sensitivity is defined as the difference in sensitivity between the current stage and the previous stage. These individuals may be detected (intercepted) by AKSO at this stage; if no screening occurs at the current stage, they will progress to the next stage.

The relevant code is publicly available at https://github.com/grailbio-publications/Hubbell_CEBP_InterceptionModel.

### List of packages and versions used in the bioinformatic analysis

R packages:

- Boruta, version 8.0.0 (used in feature selection)
- BSgenome.Hsapiens.UCSC.hg19, version 1.4.3 (used in fragmentation)
- caret, version 6.0.93 (used in model construction)
- GenomicAlignments, version 1.30.0 (used in fragmentation)
- GenomicRanges, version 1.46.1 (used in fragmentation)
- ggplot2, version 3.5.1 (used for plotting)
- ggpubr, version 0.4.0 (used for plotting)
- Rsamtools, version 2.10.0 (used in fragmentation)

Python packages:

- bitstring, version 4.3.0 (used for translating base C/T into 1/0)
- BiEntropy, version 1.1.4 (used for calculating methylation entropy)
- sklearn, version 1.2.1(used in model construction)
- matplotlib, version 3.7.2 (used for plotting)
- seaborn, version 0.11.2 (used for plotting)

### Code availability

The code used for data analysis and modeling in this study is deposited on GitHub. https://github.com/Biochain-bj/EMCED.

## Results

### Workflow of the study

**Fig. 1:**
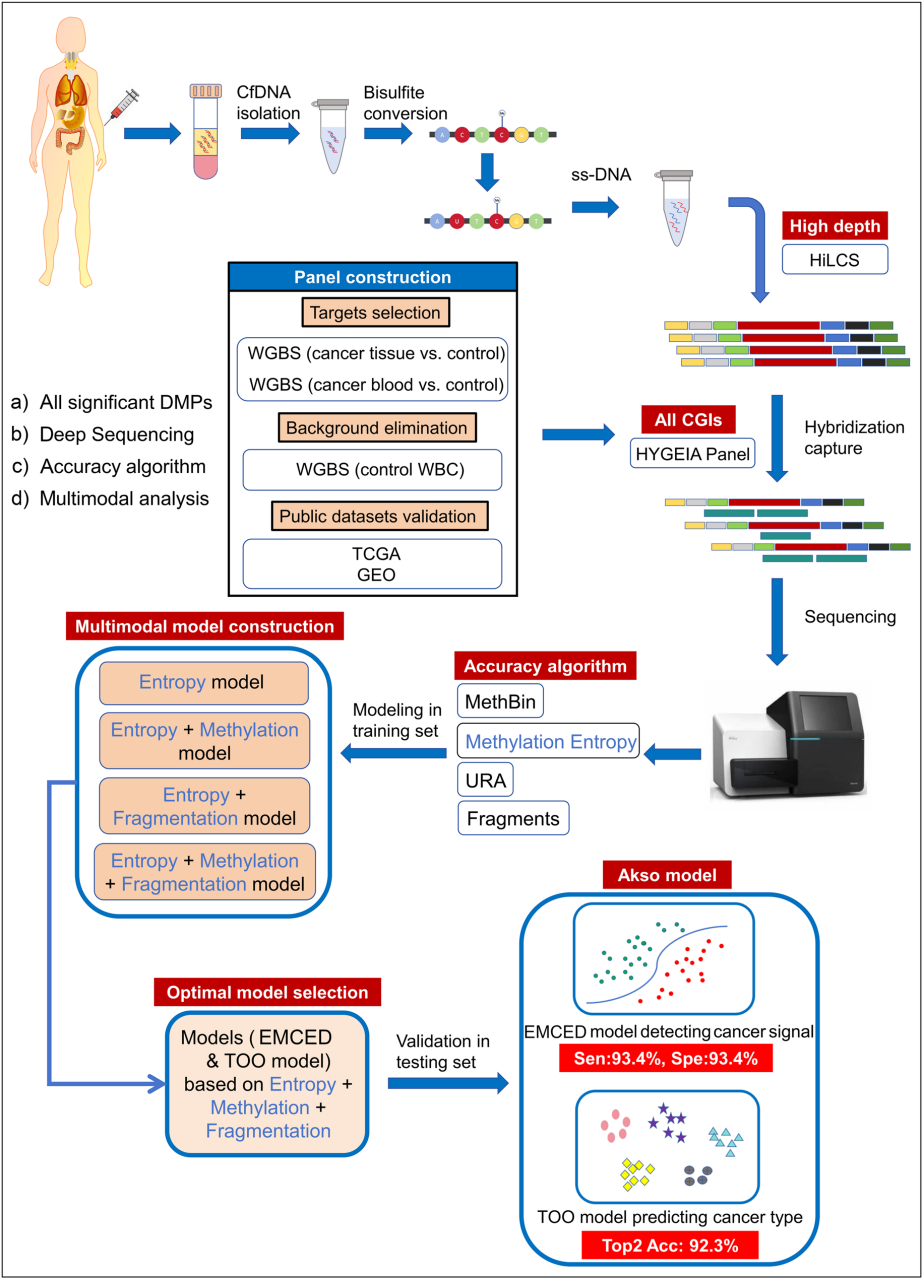
Study workflow. Schematic overview of the study illustrates the technical process from sample collection and cfDNA processing to sequencing and data analysis, presenting the key technological advantages. HiLCS, high-depth library construction system. WGBS, whole-genome methylation sequencing. WBC, white blood cell. EMCED model, Enhanced multi-cancer early detection model. TOO model, Tissue-of-Origin model. Sen, sensitivity. Spe, specificity. Acc, accuracy

The study workflow in Fig. 1 provides an overview of the key steps and methodologies employed. Before initiating blood sample analysis, we designed a customized panel (the HYGEIA panel) that integrates DNA regions derived from differential methylation analysis across multiple cancer tissues and their non-cancerous counterparts. This panel includes a comprehensive set of significant differentially methylated positions (DMPs), enabling broad coverage of epigenomic signatures. Following blood collection from subjects, cfDNA was extracted and subjected to bisulfite conversion. The converted cfDNA was then processed using a high-depth library construction system (HiLCS) to achieve ultra-deep sequencing coverage and improve the detection of rare tumor-derived fragments. The constructed libraries underwent targeted sequencing using the HYGEIA panel. Subsequent data analysis incorporated a novel computational metric, methylation entropy, to interpret cfDNA methylation patterns. Methylation entropy was computed for ~ 400 bp DNA fragments using the MethBin algorithm. Parallel analyses employing precision algorithms quantified methylation levels and fragmentation features from sequencing data. These features, methylation entropy, methylation levels, and fragmentation profiles, were integrated to develop two machine learning models: (1) a binary classification model to distinguish cancer from non-cancer (validated in an independent testing set), and (2) a Tissue-of-Origin model classifying specific cancer types. The final integrated model (the Akso model) first classifies clinical samples as cancer-positive or cancer-negative and then, for cancer-positive cases, estimates the most likely anatomical origin (Fig. 1).

### Methylation entropy calculation and analysis based on panel-targeted sequencing data of tissue samples

The HYGEIA panel was constructed at the beginning of the study and encompasses 99% of CpG islands (CGIs), as well as differentially methylated sites between various cancers and their adjacent non-cancerous tissues. Details of the panel design are presented in the section of Participants and Methods. 

**Fig. 2:**
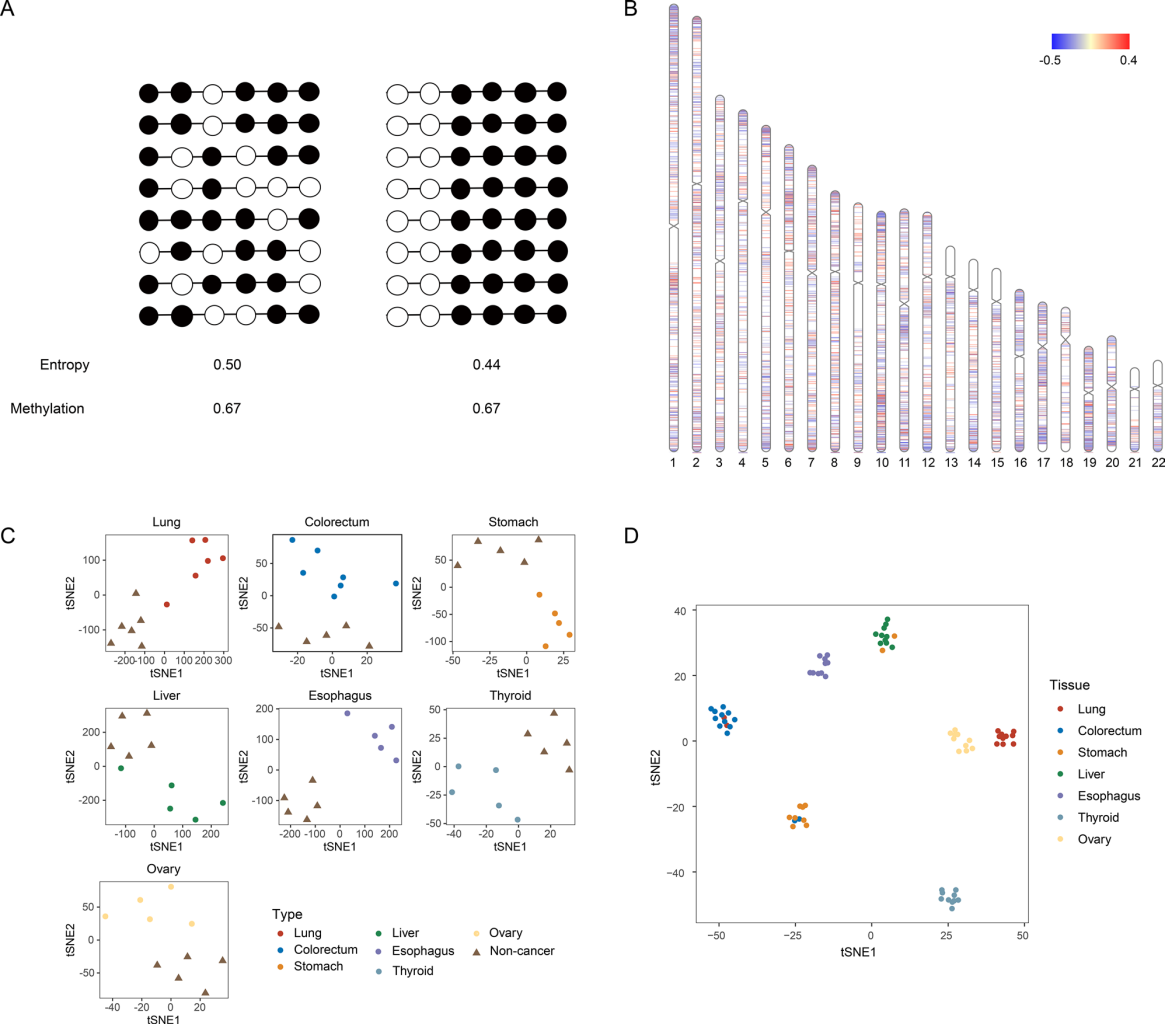
Methylation entropy-based evaluation of targeted panel performance. (**A**) Conceptual framework of methylation entropy, a novel metric quantifying methylation heterogeneity at individual DNA fragment. Black dots indicating methylated sites. White dots indicating unmethylated sites. (**B**) Genomic overview chart presenting fragment-level methylation entropy values across regions covered by the HYGEIA panel. The genomic regions with high methylation entropy in cancer tissues were labeled in red, and the low-entropy regions were labeled in blue. The color bar indicates the values corresponding to the colors, ranging from − 0.5 to 0.4. (**C**) Seven t-SNE plots indicating dimension reduction of the selected features in identifying cancer vs. non-cancer tissues based on methylation entropy values derived from panel-targeted sequencing data. (**D**) The t-SNE plot indicating dimension reduction of the selected features in differentiating 7 cancer types based on methylation entropy values derived from panel-targeted sequencing data. Each plot indicates a tissue sample

Given our interest in the role of methylation entropy in characterizing cancer-related methylation patterns, the first step of analysis focused on calculating methylation entropy for DNA fragments based on HYGEIA panel-targeted sequencing data from tissue samples. DNA fragments, divided into approximately 400 bp segments using the MethBin algorithm, were analyzed to determine methylation entropy values. Entropy values were calculated using Shannon entropy, incorporating sequencing features of methylation-site distributions. As demonstrated in Fig. 2A, when two DNA fragments exhibit identical mean methylation levels (0.67), their distinct patterns of methylation modifications result in differentiable methylation entropy values (0.50 and 0.44), highlighting entropy’s superior ability to capture methylation variation. The genomic overview of methylation entropy illustrates regions across the genome with increased (red) or decreased (blue) entropy in cancer tissues relative to non-cancer tissues (Fig. 2B).

Based on the observed differences in methylation entropy between cancer and non-cancer tissues, we hypothesized that these sample types would exhibit distinct methylation entropy profiles. Thus, we performed dimension reduction and clustering analyses for fragment-level methylation entropy values of each tissue sample and visualized by t-SNE plots, which revealed that each cancer tissue and its corresponding adjacent normal tissue were localized to distinct regions (Fig. 2C). Similarly, samples from patients with seven cancer types were almost clustered separately (Fig. 2D), suggesting the discriminatory power of methylation entropy for original tissues.

### Construction the enhanced multi-cancer early detection model (EMCED model) and the performance testing

**Fig. 3:**
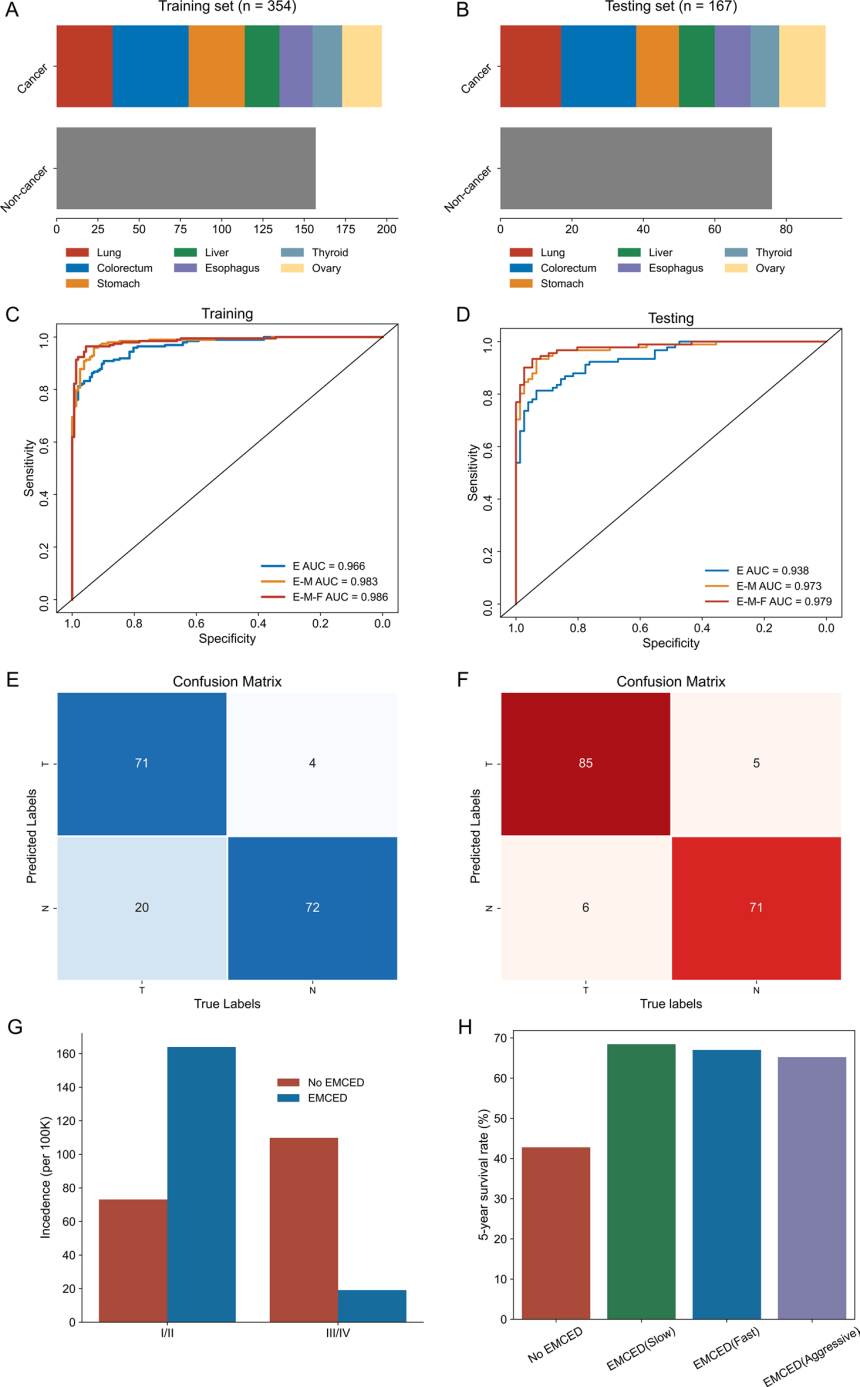
EMCED model construction and potential clinical benefit prediction. (**A**, **B**) Case distribution of the seven cancer types and the control in training (**A**) and testing (**B**) sets. (**C**, **D**) The ROC curves of the binary classification models based on methylation entropy, methylation entropy + methylation levels, methylation entropy + methylation levels + fragmentation features respectively, in training (**C**) and testing (**D**) sets. (**E**) The confusion matrix displaying the detection results of the binary classification model based on methylation entropy in testing set. (**F**) The confusion matrix displaying the detection results of the binary classification model based on methylation entropy + methylation levels + fragmentation features in testing set. (**G**, **H**) Simulating real-world estimation indicating the variations in stage distribution (**G**) and in 5-year survival rate (**H**) with and without employing EMCED model. The group employed EMCED were stratified into three subgroups, slow, fast and aggressive, according to the progression rate of cancer

Based on the above findings, we aimed to construct a binary classification model to distinguish cancer from non-cancer samples. To develop the models, clinical blood samples (*n* = 521) were collected from the Affiliated Cancer Hospital of Inner Mongolia Medical University. Among these samples, the 288 cancer samples comprised 67 cases of colorectal cancer, 30 cases of esophageal cancer, 31 cases of hepatocellular cancer, 51 cases of lung cancer, 46 cases of gastric cancer, 26 cases of thyroid cancer, and 37 cases of ovarian cancer, and the control samples were derived from 233 non-cancer individuals. The clinical cohort were divided into training and testing datasets, with participants’ clinical characteristics summarized in Table 1 and groups distribution displayed in Fig. 3A and B. The detailed case numbers for each cancer type are presented in Supplementary Table S1.

**Table 1:** Baseline characteristics of the participants

Variable	Training set		Testing set	
	Cancer (*n* = 197)	Non-cancer (*n* = 157)	Cancer (*n* = 91)	Non-cancer (*n* = 76)
Age, mean (± SD)	59 (± 12)	51 (± 9)	60 (± 11)	50 (± 7)
Sex, n (proportion)				
Male	96 (48.7%)	90 (57.3%)	45 (49.5%)	46 (60.5%)
Female	101 (51.3%)	67 (42.7%)	46 (50.5%)	30 (39.5%)
TNM stage, n (proportion)				
I	41 (20.8%)		20 (22.0%)	
II	45 (22.8%)		21 (23.1%)	
III	64 (32.5%)		28 (30.8%)	
IV	13 (6.6%)		7 (7.7%)	
Unknown	34 (17.3%)		15 (16.5%)	

SD, standard deviation. TNM, tumor-node-metastasis

After extracting cfDNA from these clinical blood samples and performing panel-targeted sequencing, we analyzed the data to calculate fragment-level methylation entropy values. Next, a binary classification model based on methylation entropy (E model) was developed, yielding AUC values of 0.966 in the training set (Fig. 3C) and 0.938 in the testing set (Fig. 3D). Moreover, five-fold cross-validation further confirmed the stability of the entropy model’s performance (Supplementary Figure S1). Building upon this model, we incorporated additional features, including methylation levels and fragmentation features, into subsequent models. The receiver operating characteristic (ROC) curves also illustrate the comparative performance of the model combining methylation entropy and methylation levels (E-M model) and the model integrating entropy, methylation levels, and fragmentation features (E-M-F model) in both the training set (Fig. 3C) and the testing set (Fig. 3D), with corresponding AUC values indicated. Further evaluation of each model in the testing set, including AUC, sensitivity, and specificity, is summarized in Table 2. These results indicate that the integrated E-M-F model achieved the highest overall performance, with both sensitivity and specificity reaching 93.4%. Meanwhile, we also present the entire model construction process in the form of a schematic flowchart in Supplementary figure S2. The confusion matrices for the E model and E-M-F model compare the estimates of each model to the true labels determined by reference methods (Fig. 3E and F). The E-M-F model demonstrated an increased number of true-positive cases compared with the E model, while the number of true-negative cases remained nearly unchanged. Based on these findings, we selected the E-M-F model as our final classifier for distinguishing cancer from non-cancer using cfDNA panel-targeted sequencing data and designated it as the Enhanced Multi-Cancer Early Detection model (EMCED model). We further assessed the overall sensitivity of the EMCED model across seven cancer types, as well as its sensitivity in early-stage (Stage I-II) cancers, with the results summarized in Table 3. Some cancer types exhibited an apparent sensitivity of 100%. However, these estimates should be interpreted in the context of the corresponding sample sizes, which are reported in the first column of the table. In particular, 100% sensitivity for early stage (Stage I–II) in certain cancers is most likely a consequence of the limited sample sizes when stratifying simultaneously by cancer type and stage. The detailed numbers of cases for each cancer type and stage, in both the training and testing sets, are provided in Supplementary Table S1.

Because there was some age imbalance between cancer and non-cancer groups (Table 1), we evaluated age as a potential confounder by adding it as a feature to the methylation entropy model (E), methylation level model (M), fragmentation model (F), and the combined E–M–F model. As shown in Supplementary Figure S3, including age produced only minimal changes in ROC curves in both training and testing sets; for the final E–M–F model, the ROC curve with age was almost identical to that of the original EMCED model (Supplementary Figure S3D). Supplementary Table S2 further shows that performance metrics were largely unchanged, indicating that discrimination between cancer and non-cancer is not driven by age. These results support that the contribution of methylation entropy and other methylation features is largely independent of age, and that age is not driving the observed discrimination between cancer and non-cancer. For each model, we also computed a score for every sample and examined the correlation between age and model scores. Consistently, model scores were essentially uncorrelated with age (*R* < 0.2, *p* > 0.05 for all models; Supplementary Figure S4A–D). further indicating that the model features separating cancer from non-cancer are not proxies for age. We also compared scores between males and females (Supplementary Figure S5A–D) and found no significant sex-related differences, suggesting that sex is likewise not a major driver of the predictive signal.

**Table 2:** Performance evaluation of binary classification models in testing set

Models	AUC value	Sensitivity (95% CI)	Specificity (95% CI)
E model	0.938	78.0% (68.1%-86.0%)	94.7% (87.1%-98.5%)
E-M model	0.973	91.2% (83.4%-96.1%)	93.4% (85.3%-97.8%)
E-M-F model	0.979	93.4% (86.2%-97.5%)	93.4% (85.3%-97.8%)

E, methylation entropy. M, methylation level. F, fragmentation feature

**Table 3:** Sensitivity assessment of EMCED model across seven cancer types in testing set

Cancer types (Sample volume, *n*)	Sensitivity (95% CI)	Early-stage (I-II) sensitivity (95% CI)
Colorectum (21)	90.5% (69.6%-98.8%)	80.0% (28.4%-99.5%)
Lung (17)	94.1% (71.3%-99.9%)	91.7% (61.5%-99.8%)
Stomach (12)	100.0% (73.5%-100.0%)	100.0% (39.8%-100.0%)
Ovary (13)	76.9% (46.2%-95.0%)	50.0% (11.8%-88.2%)
Liver (10)	100.0% (69.2%-100.0%)	100.0% (29.2%-100.0%)
Esophageal (10)	100.0% (69.2%-100.0%)	100.0% (29.2%-100.0%)
Thyroid (8)	100.0% (63.1%-100.0%)	100.0% (63.1%-100.0%)

Additionally, based on the incidence and stage distribution of multiple cancers in China, we performed real-world performance simulations for the EMCED model. The results indicated that implementation of the EMCED model could shift the stage distribution ratio from 73/110 (stage I/II to III/IV) to 162/19, substantially increasing the proportion of cancer detected at curable stages and improving cost-effectiveness (Fig. 3G). Due to stage shift, the 5-year survival rate would increase from 43% to over 60% across three scenarios defined by different cancer progression rate (Fig. 3H). These results suggest the potential benefits of the EMCED model for population-level cancer screening, including earlier detection, improved clinical outcomes, and enhanced disease management.

### Construction and performance evaluation of the tissue-of-origin (TOO) model

**Fig. 4:**
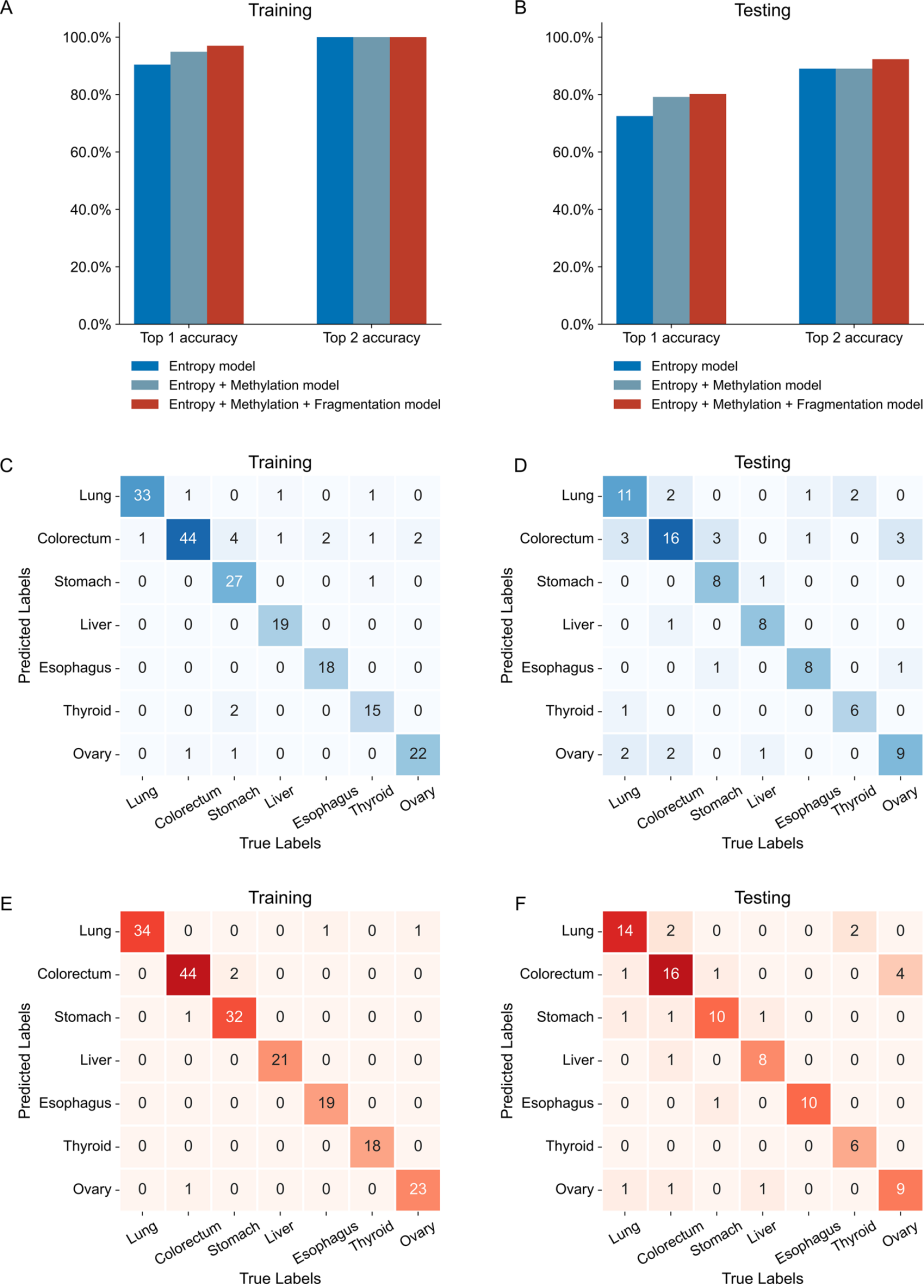
Tissue-of-origin (TOO) model construction and performance evaluation. (**A**, **B**) The TOP1 accuracy (left) and the TOP2 accuracy (right) of the TOO models based on methylation entropy, methylation entropy + methylation levels, methylation entropy + methylation levels + fragmentation features respectively, in training (**A**) and testing (**B**) sets. (**C**, **D**) Confusion matrices displaying the TOP1 tissue-location results of the TOO model based on methylation entropy in training (**C**) and testing (**D**) sets. (**E**, **F**) Results of the TOO model based on methylation entropy + methylation levels + fragmentation features in training (**E**) and testing (**F**) sets

Distinct methylation entropy profiles observed among different cancer types were leveraged to build a tissue-of-origin model for classifying cancer types. The performance of the TOO model based on entropy solely underwent comprehensive evaluation across both training and testing datasets. Specifically, the model’s accuracy was assessed by computing two metrics: TOP1, representing the most probable cancer type, and TOP2, encompassing the two most probable cancer types (Fig. 4A and B). In the testing set, the methylation entropy TOO model achieved a TOP1 accuracy of 72.5% and a TOP2 accuracy of 89.0% (Fig. 4B). To further enhance tissue-of-origin classification performance, we developed a composite model by integrating additional features, including methylation levels and fragmentation features, alongside methylation entropy (E-M-F TOO model). This integrated approach improved the TOP1 accuracy to 80.2% and the TOP2 accuracy to 92.3% in the testing set, representing the highest tissue-of-origin classification performance among three models displayed in Fig. 4B. The performance of the methylation entropy and methylation level-based TOO model (E-M TOO model) generally falls between the entropy-only and E-M-F TOO models (Fig. 4A and B).

Confusion matrices illustrating the performance of the methylation entropy TOO model (Fig. 4C and D) and the E-M-F TOO model (Fig. 4E and F) compare the predicted cancer types with the reference detection methods. The numbers along the diagonal of each matrix represent the count of samples for which the model’s estimates were consistent with the reference detection methods. Both the methylation entropy TOO model and the E-M-F TOO model demonstrated high concordance with the reference method in the training set. Notably, the E-M-F TOO model exhibited superior tissue-of-origin classification effects in the testing set. Corresponding confusion matrices in the supplementary materials show the comparison of model’s estimates to the reference methods for the methylation level-based TOO model (Supplementary Figure S6A and S6B) and the fragmentation feature-based TOO model (Supplementary Figure S6C and S6D). In terms of the number of samples in agreement with the reference methods, these two TOO models presented in the supplementary did not perform as well as the E-M-F TOO model.

## Discussion

In this study, we performed a multidimensional analysis of biomarkers for multi-cancer detection and developed a pan-cancer screening model with strong clinical application potential. Notably, we introduced a novel dimension for cancer detection by leveraging methylation entropy, a methodological innovation that, to our knowledge, has not been reported previously. Integrating the Tissue-of-Origin model with the binary classification model substantially expands the potential clinical applicability and translational value of our approach. Our multi-modal analysis of genomic methylation data also offers new clinical research perspectives and provides novel analytical strategies for future methylation-based research. The results from both the EMCED model and the TOO model clearly demonstrate significant differences in methylation entropy between cancer and non-cancer samples, while distinct entropy signatures were also observed among individual cancer types. Specifically, regions with characteristic methylation entropy profiles differentiated cancer from non-cancer samples, and different cancer types also exhibited their own methylation entropy patterns.

Throughout model development and validation, our study cohort comprised 521 enrolled participants, including 288 patients spanning seven cancer types and 233 non-cancer controls. In order to emphasize generalizability, subjects were stratified by age, sex, cancer type, and clinical stage, then randomly allocated to a training set and a testing set, with clinical characteristics balanced between the training and testing sets.

It is worth noting that, the workflow employed in the study highlights methodological strengths in identifying cancer-specific methylation patterns. A key advance of our approach is the synergistic coupling of an extensive informed capture panel with ultra-high-depth library preparation, which together overcome the intrinsic challenges of low-abundance ctDNA in plasma [24, 25]. The customized panel, the HYGEIA panel, was constructed by systematically integrating DNA methylation data from multiple high-quality sources, including WGBS datasets from carcinoma, adjacent normal tissues, peripheral blood, and comprehensive data from the TCGA and GEO cohorts. By extensively and deeply mining whole-genome data, the HYGEIA panel ensures broad yet selective coverage of most genomic CpG sites, including differentially methylated positions (DMPs) and dense regions within CpG islands, shores, and shelves. These captured CpG sites also comprehensively encompass promoter and gene body regions involved in key cancer pathways. Furthermore, the employment of the HiLCS (High-depth Library Construction System) technique for plasma cfDNA delivers another major advantage, yielding high sequencing depth (mean depth > 200X). This technological superiority ensures that even sparse tumor-derived fragments carry sufficient read support for reliable entropy estimation, maximizing the likelihood of blood-sampling detection for relevant information.

The combination of the expansive and well-curated HYGEIA panel with ultra-deep sequencing enables highly sensitive detection of heterogeneous methylation patterns associated with cancer. Given that peripheral ctDNA typically represents only a small proportions of total cfDNA [24], and its release into the bloodstream is limited in early-stage cancers [26], our panel design strategically incorporated methylation profiles from a wide array of cancer tissue samples and maximized the inclusion of genomic regions and loci most relevant for cancer detection. After collecting cfDNA samples from our study cohort, we applied the HiLCS library construction technique to achieve ultra-deep sequencing coverage, thereby enriching rare tumor-derived methylation signatures within the libraries. Subsequent deep sequencing using the HYGEIA panel allowed for deep exploration of valuable methylation information in cfDNA, minimizing the loss of potentially malignant signals. This integrative approach systematically combines broad genomic capture with in-depth mining, meeting the unique demands of ctDNA methylation analysis.

In practical implementation, we generally consider 10 ng of cfDNA as the minimum input required to achieve reliable methylation profiling performance. In this study, the lowest cfDNA amount used for library preparation among all analyzed plasma samples was 10.36 ng, which still produced robust analytical results. This input level is comparable with previously reported cfDNA requirements in large‑scale studies, such as the CCGA study (approximately 75 ng per assay) and the THUNDER study (5–30 ng) [27, 28], supporting the technical robustness and feasibility of our assay for clinical application.

Notably, this study introduced an innovative approach by not only calculating methylation entropy from panel-targeted sequencing data of peripheral cfDNA, but also leveraging these entropy features to develop the cancer detection model. In order to quantify the characteristics of cfDNA methylation patterns, we adapted the BiEntropy framework [21] to each fragment of cfDNA sequencing data. This approach captures not only the overall proportion of methylated CpGs but also the degree of local patterning that simple mean-methylation levels cannot resolve. Conceptually, methylation entropy here reflects the disorder or heterogeneity of CpG methylation states along a genomic segment: high entropy indicates a mosaic of methylated and unmethylated sites in corresponding fragment, whereas low entropy indicates more uniform methylation patterns. The choice of fragment size (300–400 bp) balances the need for sufficient CpG density to estimate entropy against the desire to localize epigenetic variability to genomic loci. Elevated methylation entropy in cancer samples likely arises from the well-documented epigenomic instability and clonal heterogeneity of tumors, whereas normal tissues tend to maintain more orderly methylation landscapes [29–31]. Our results demonstrate that fragment-level methylation entropy serves as a robust discriminator of cancer versus non-cancer cfDNA and, when combined with methylation levels and fragmentation features, further improves the performance of classification.

The EMCED and TOO model developed in this study demonstrated robust performance in multicancer detection. In the testing set, the EMCED model achieved 93.4% sensitivity and specificity overall, with sensitivities exceeding 70% across seven cancer types (six types > 90%). The TOO model exhibited the TOP2 tissue-of-origin accuracy of 92.3%. Compared with previous studies, our binary classifier achieved an overall sensitivity of 93.4% and an overall specificity of 93.4%. In the THUNDER study, sensitivity was 69.1% at a specificity of 98.9%, and 75.1% at a specificity of 95.1%. The CCGA study reported an overall sensitivity of 51.5% and a specificity of 99.5% [27, 28, 32]. While our classifier demonstrates slightly lower specificity than these published studies, it provides higher sensitivity, which may be attributed to the incorporation of methylation entropy as an additional feature in our model. However, the THUNDER study and the CCGA study utilized large independent cohorts to evaluate model performance [28, 32], underscoring the necessity for expanded validation in future research to strengthen clinical applicability.

In the EMCED model, we observed heterogeneous sensitivities across the seven cancer types. This likely reflects underlying biological differences between these cancers. Different tumors shed cfDNA with distinct methylation landscapes that mirror their tissue of origin, cell type, molecular pathways, and epigenetic reprogramming. Our cohort included gastrointestinal cancers (esophageal, gastric, colorectal), hepatocellular carcinoma, lung cancer, thyroid cancer, and ovarian cancer, which arise from diverse epithelial, parenchymal, and endocrine‑related cells. Such diversity can lead to cancer‑specific methylation entropy patterns and variable detectability. Methylation entropy captures disruption of methylation patterns, which may be greater in more aggressive or genomically unstable tumors. Besides, ctDNA shedding kinetics also differ by cancer type. Colorectal, esophageal, and liver cancers are generally considered to have relatively high shedding, making their signals easier to separate from background noise [33]. The 100% early-stage sensitivity observed for gastric, esophageal and liver cancers, in our study may therefore reflect both biological features and the limited sample size. Nevertheless, similarly high performance for specific cancers has also been reported in large multi‑cancer methylation studies, such as CCGA Study 3, where the sensitivity was 93.5% for liver cancer and the detection rate for ovarian cancer was even higher than in our study, reaching 83.1% [32].

Considering the clinical application, this study prospectively collected blood samples and applied a blinded analytical process, simulating the real‑world clinical application of such cancer detection models, in which clinicians, laboratory staff, and data analysts remain unaware of the final diagnostic outcome at the time of blood sample collection. Therefore, we envision EMCED/TOO primarily as an initial blood-based detection approach in clinical context. After clinical consultation and basic examinations, cfDNA methylation testing could be integrated with standard-of-care results to help determine next-step investigations (e.g., targeted imaging or endoscopy), which complements existing diagnostic workflows. This strategy could potentially reduce unnecessary invasive procedures in patients with suspected but non-malignant lesions. This is particularly relevant because performance and applicability vary widely across current modalities. For lung cancer, low-dose CT (LDCT) screening generally shows high sensitivity but high false-positive rate (baseline false-positive rates in the range of roughly 20–30%) [34]. For colorectal cancer, FIT provides a noninvasive option but has an average sensitivity of 79% and requires follow-up colonoscopy [35]. For liver cancer surveillance, ultrasound has limited sensitivity for early-stage HCC and even combined with AFP can only achieve an early-stage sensitivity of 63% [36]. For several cancers that rely heavily on imaging-based early detection (e.g., ovarian and thyroid), imaging findings may not reliably distinguish benign from malignant lesions [37, 38]. In addition, the detection of gastric cancer and esophageal cancer mainly relies on endoscopy, and there is currently no widely recognized non-invasive early screening method. In comparison, EMCED achieved sensitivity and specificity > 90% in our testing set, suggesting that the model may provide clinically useful complementary information across cancer types. Furthermore, the model may also have potential applications in treatment monitoring and recurrence surveillance.

On the other hand, given the strong performance of EMCED/TOO models, they also hold promise as a potential tool for future blood-based cancer screening. In a screening context, such an assay could potentially serve as an alternative to certain single-cancer tests and offers practical advantages over running multiple organ-specific screens separately: a single blood draw can simultaneously evaluate multiple cancers, enabling a more streamlined workflow, improving convenience and potentially adherence. From a health economics perspective, screening for a single cancer type may yield limited benefit, particularly for cancers with low incidence, whereas multi-cancer screening can offer clearer advantages in terms of cost-effectiveness. Nonetheless, screening use will require prospective evaluation in general-risk populations.

### Limitations

A major limitation of the study is the relatively small sample size. Although the cohort provides sufficient power for exploratory analyses, the limited number of participants in testing cohort constrains the generalizability of our findings. In several cancer types, the seemingly perfect (100%) sensitivity estimates are driven by very small denominators, as reflected by the wide 95% confidence intervals, and therefore should be interpreted with caution. These findings underscore the need for larger, independent validation cohorts to obtain more precise sensitivity estimates. As a single-center study, the cohort may not fully capture the heterogeneity present across broader populations. Future multi-center studies involving larger cohorts and participants from diverse geographic and ethnic backgrounds, as illustrated by large-scale clinical trials such as the R. Zhang et al.’s study [39], will be essential to further independent validate and refine our models. In addition, there was some age imbalance between the cancer and non-cancer groups in the model construction cohort of this study. Although we applied multiple analytical strategies and showed that this age imbalance had no substantial impact, it nevertheless represents a potential source of bias that should be minimized. Therefore, in future studies, we will strive to more closely match the age distribution between case and control groups. Such efforts will help to enhance the robustness of methylation entropy-involved cancer detection model, and ensure that the model performance can be reliably extended to a variety of clinical settings and populations.

## Conclusions

Our study demonstrates that multidimensional analysis of cfDNA methylation features, particularly incorporating methylation entropy, enables sensitive and robust detection of multiple cancer types and effective tissue-of-origin classification. The integration of novel analytical dimensions and advanced technologies has substantial potential to advance non-invasive pan-cancer detection, laying a strong foundation for the clinical translation of this approach and highlighting its promise for improving early cancer screening and disease management.

## Supplementary Information

Below is the link to the electronic supplementary material.

Supplementary Material 1

## Data Availability

The data analyzed in this study are available upon reasonable request from the corresponding author.
