# Supplementary material for: Methylation entropy as a novel dimension in liquid biopsy: enhanced multimodal framework for cancer detection and tissue-of-origin classification
Source: J Transl Med. 2026 Mar 19;24:597. doi: 10.1186/s12967-026-08028-x (PMC13122931; doi:10.1186/s12967-026-08028-x)
Supplement: Supplementary file 1 — Supplementary Material 1 [file 12967_2026_8028_MOESM1_ESM.pdf]

Supplementary Figures and Tables

Supplementary Figure S1

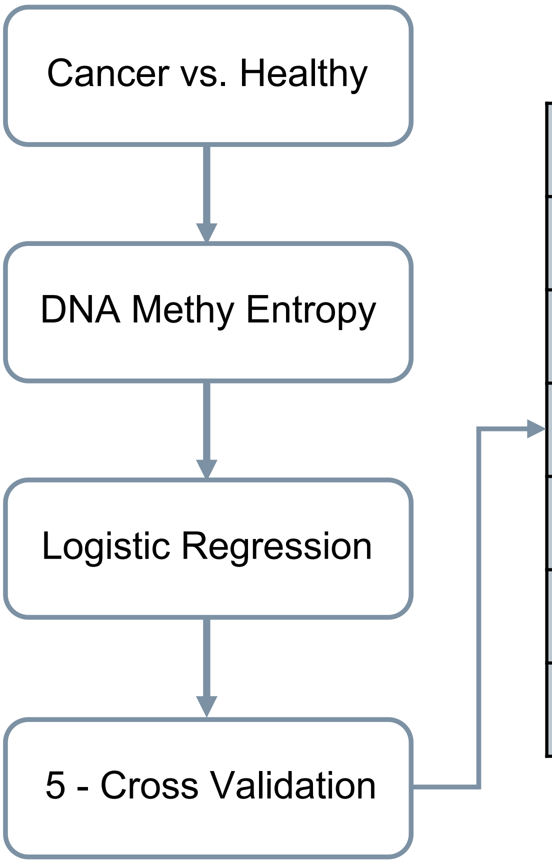

CV	Training	Testing
CV1	0.962	0.937
CV2	0.922	0.939
CV3	0.935	0.904
CV4	0.956	0.854
CV5	0.941	0.937
Mean	0.943	0.914

Supplementary Figure S1. Five-fold cross-validation analysis of the entropy-based binary classification model. The area under curve (AUC) values for each fold in both the training and testing datasets demonstrate the stability and robustness of the model's performance across multiple validation sets.

Supplementary Figure S2

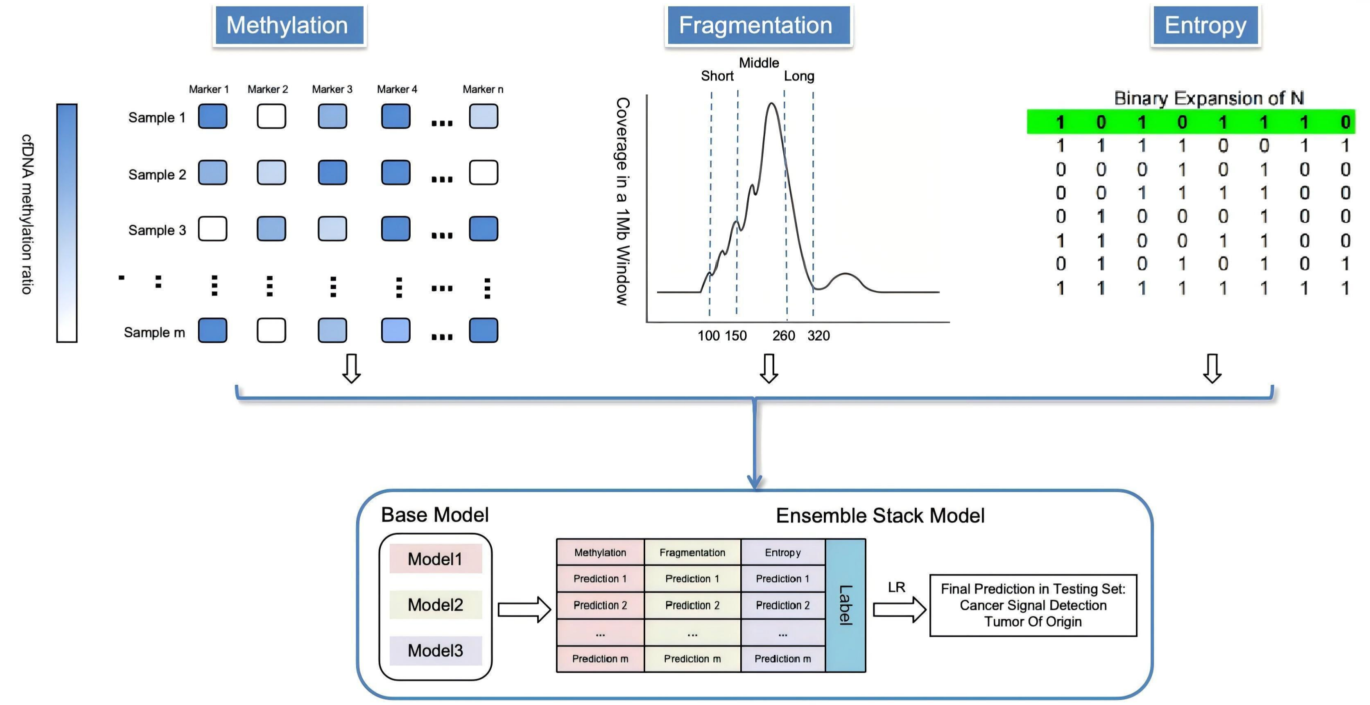

Supplementary Figure S2. Schematic of the Proposed Model Construction and Inference Pipeline.

Supplementary Figure S3

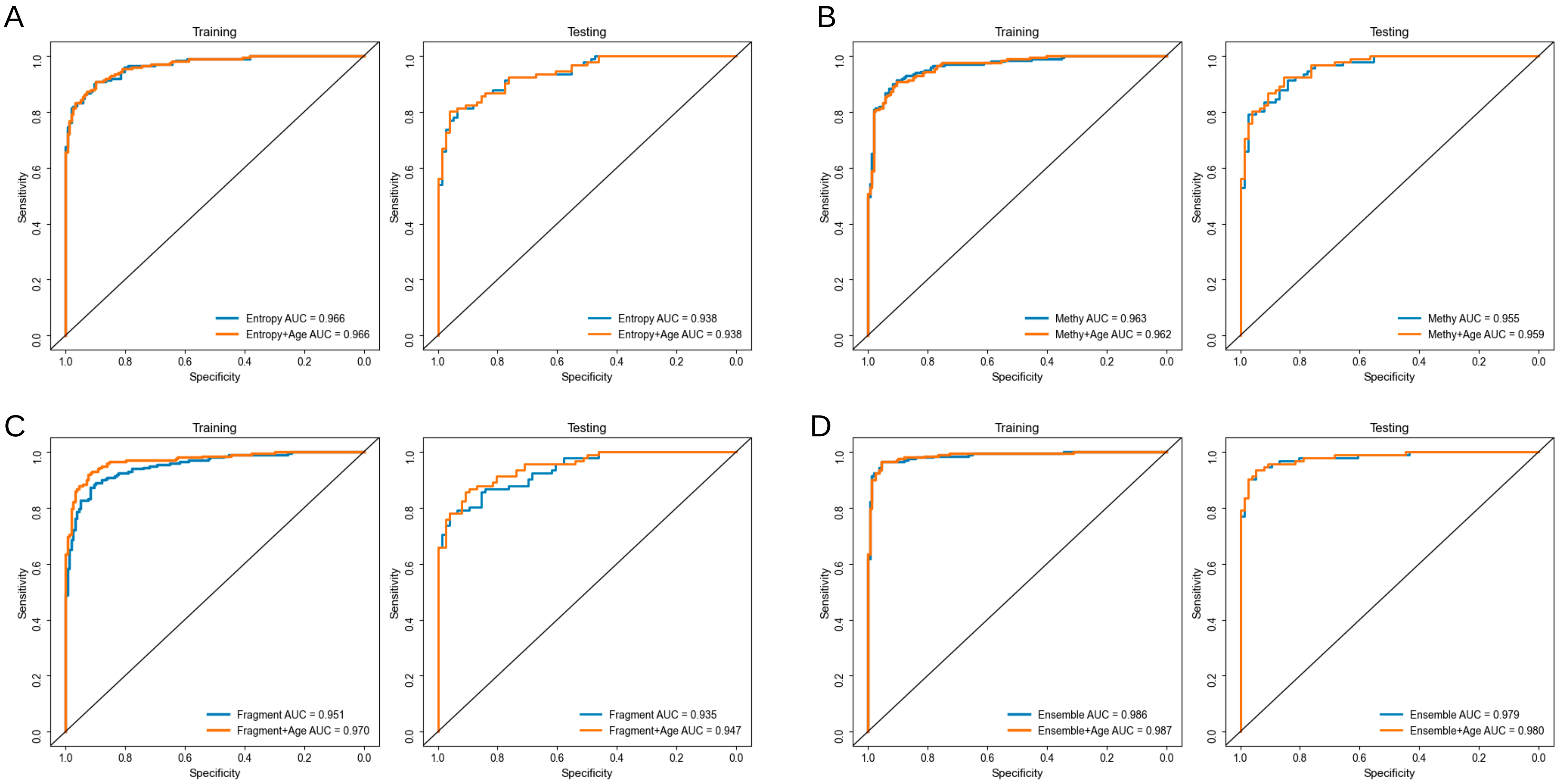

Supplementary Figure S3. ROC curves for binary classifiers distinguishing cancer from non-cancer after incorporating age as an additional feature. (A) Methylation entropy– based model; (B) methylation level– based model; (C) fragmentation feature– based model; (D) integrated model combining methylation entropy, methylation level, and fragmentation features. Blue curves indicate the original models, and orange curves indicate the corresponding models with age included. The AUC values are shown in each plot.

Supplementary Figure S4

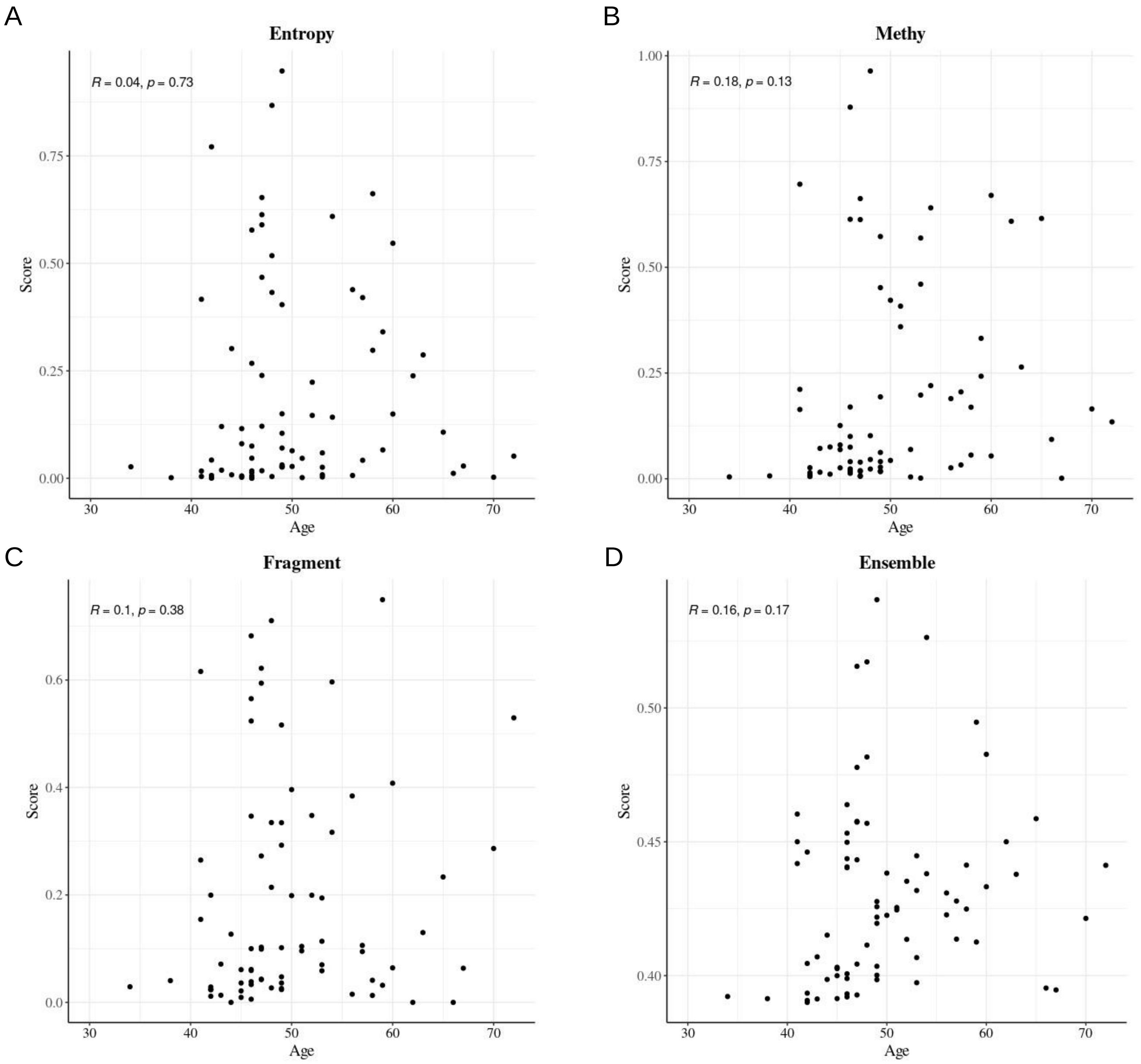

Supplementary Figure S4. Correlation analysis between model score and age. (A) Entropy model score. (B) Methylation level model score. (C) Fragmentation model score. (D) E-M-F model score. R, correlation coefficient. p, p value. Sample: testing set.

Supplementary Figure S5

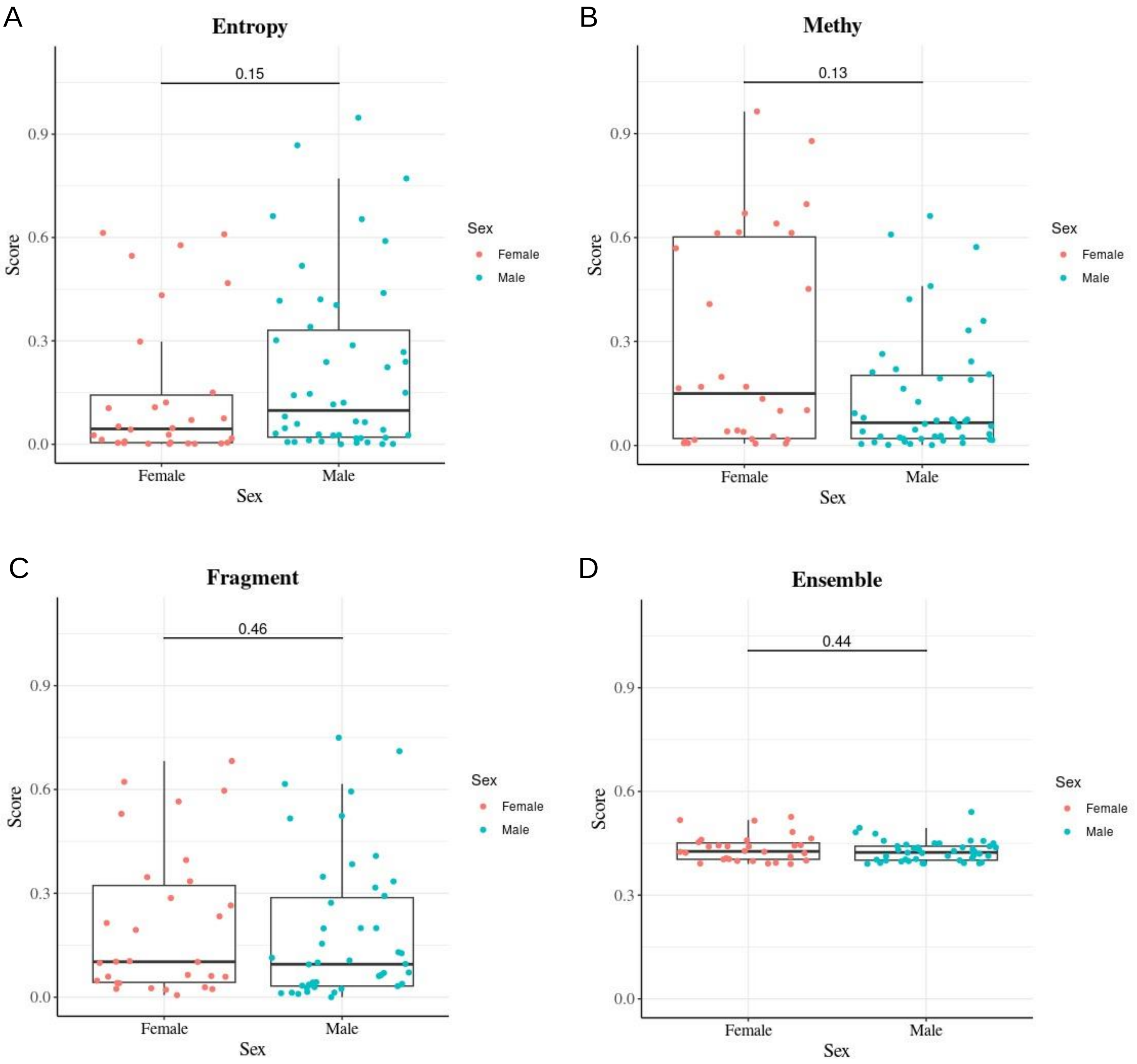

Supplementary Figure S5. Analysis of model score differences between males and females. (A) Entropy model score. (B) Methylation level model score. (C) Fragmentation model score. (D) E-M-F model score. P values derived from t-tests were shown on the plots. Sample: testing set.

Supplementary Figure S6

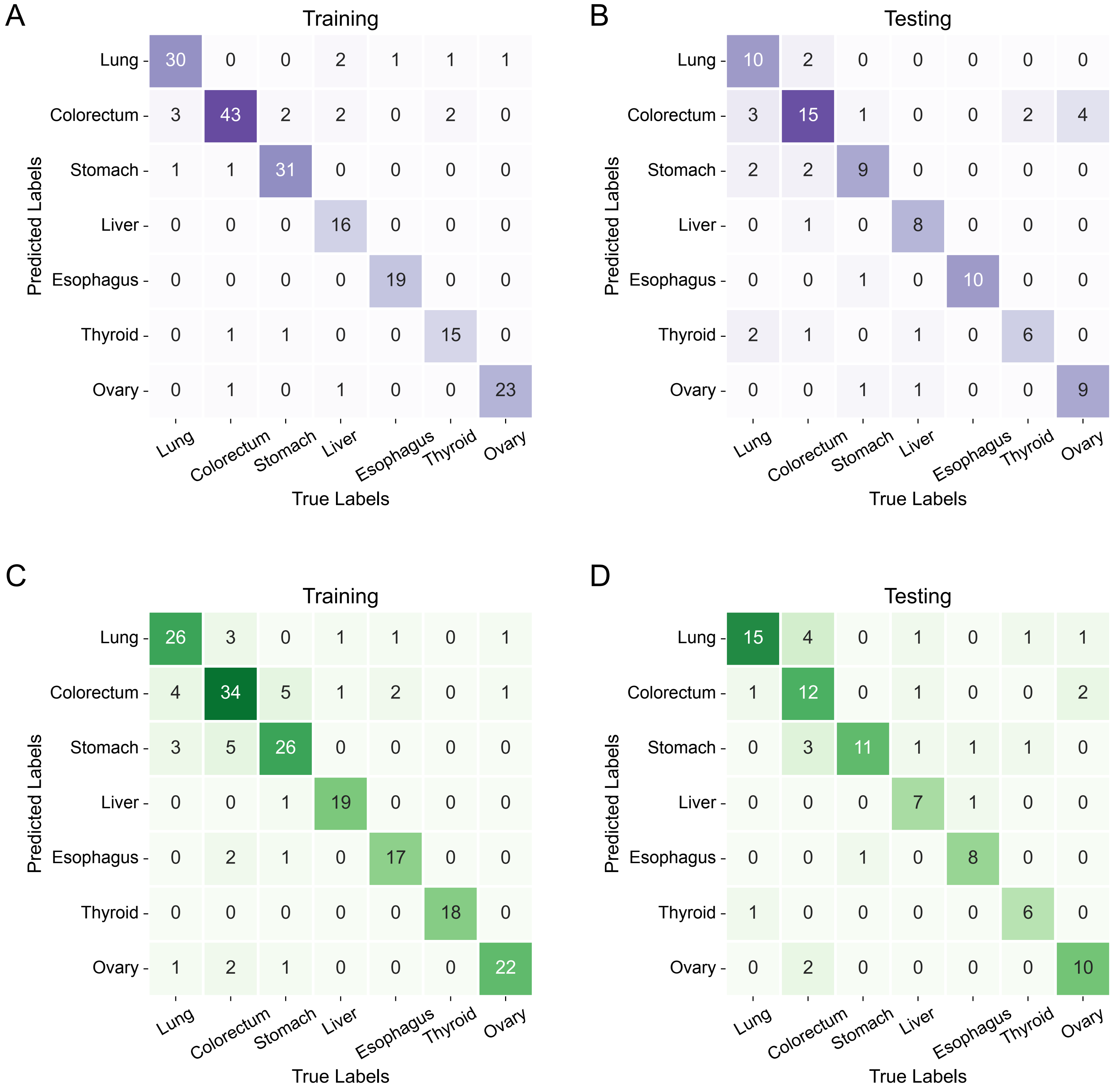

Supplementary Figure S6. Performance evaluation of other two tissue-of-origin models based on single feature. (A, B) Confusion matrices displaying the TOP1 tissue-location prediction results of the TOO model based on methylation levels in training (A) and testing (B) sets. **(C, D)** Confusion matrices displaying the TOP1 tissue-location prediction results of the TOO model based on fragmentation features in training (C) and testing (D) sets.

Supplementary Table S1. Case numbers of each cancer type in training and testing sets.

Cancer type	Case numbers								Total sample volume
	Training set (n = 197)				Testing set (n = 91)				
	Early stage (I-II)	Late stage (III-IV)	Unknown	Total number	Early stage (I-II)	Late stage (III-IV)	Unknown	Total number	
Colorectum	8	25	13	46	5	9	7	21	67
Lung	24	8	2	34	12	4	1	17	51
Stomach	18	15	1	34	4	8	0	12	46
Ovary	7	17	0	24	6	7	0	13	37
Liver	7	5	9	21	3	4	3	10	31
Esophageal	5	6	9	20	3	3	4	10	30
Thyroid	17	1	0	18	8	0	0	8	26

Supplementary Table S2. Performance metrics for binary classifiers after incorporating age as an additional feature (in testing set).

Models	Feature without Age	Feature + Age
E model	Sen=0.780，Spe=0.947，AUC=0.938	Sen=0.769，Spe=0.961，AUC=0.938
M model	Sen=0.791，Spe=0.974，AUC=0.955	Sen=0.802，Spe=0.947，AUC=0.959
F model	Sen=0.747，Spe=0.961，AUC=0.935	Sen=0.780，Spe=0.921，AUC=0.947
E-M-F model	Sen=0.934，Spe=0.934，AUC=0.979	Sen=0.945，Spe=0.921，AUC=0.980

Sen, sensitivity. Spe, specificity. AUC, area under curve value. The cutoff value is selected based on the maximum Youden index to determine sensitivity and specificity.
